# A Rapid Extraction Method Combined with a Monoclonal Antibody-Based Immunoassay for the Detection of Amatoxins

**DOI:** 10.3390/toxins11120724

**Published:** 2019-12-11

**Authors:** Candace S. Bever, Robert M. Hnasko, Luisa W. Cheng, Larry H. Stanker

**Affiliations:** 1 Foodborne Toxin Detection and Prevention Unit, Western Regional Research Center, Agricultural Research Service, United States Department of Agriculture, 800 Buchanan Street, Albany, CA 94710, USA; 2 Produce Safety and Microbiology Unit, Western Regional Research Center, Agricultural Research Service, United States Department of Agriculture, 800 Buchanan Street, Albany, CA 94710, USA

**Keywords:** amatoxins, amanitins, immunoassay, monoclonal antibodies, ELISA, death cap mushrooms

## Abstract

Amatoxins (AMAs) are lethal toxins found in a variety of mushroom species. Detection methods are needed to determine the occurrence of AMAs in mushroom species suspected in mushroom poisonings. In this manuscript, we report the generation of novel monoclonal antibodies (mAbs, AMA9G3 and AMA9C12) and the development of a competitive, enzyme-linked immunosorbent assay (cELISA) that is sensitive at 1 ng mL^−1^ and shows selectivity for α-amanitin (α-AMA) and γ-amanitin (γ-AMA), and less for β-amanitin (β-AMA). In order to decrease the overall time needed for analysis, the extraction procedure for mushrooms was also simplified. A rapid (1 min) extraction procedure of AMAs using solvents as simple as water alone was successfully demonstrated using *Amanita* mushrooms. Together, the extraction method and the mAb-based ELISA represent a simple and rapid method that readily detects AMAs extracted from mushroom samples.

## 1. Introduction

**Figure 1:**
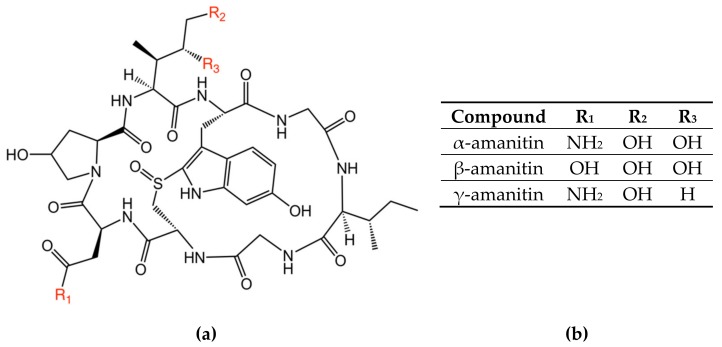
Chemical structures of the amatoxin variants examined in this paper, (**a**) molecular structure of amanitin, (**b**) R-group designations for each variant.

There are thousands of reported mushroom poisonings occurring worldwide each year [1,2,3,4,5]. The most severe cases are from amatoxin (AMA)-containing mushrooms. AMA-containing mushrooms include a few species from the genera *Amanita*, *Galerina*, and *Lepiota*. The principle toxins responsible for the poisonings are the bicyclic octapeptides known as AMAs, most notably α-amanitin (α-AMA) and β-amanitin (β-AMA), and possibly γ-amanitin (γ-AMA) (Figure 1). In mice, the LD_50_ for α-AMA is 0.1 mg kg^−1^ [6] and, to humans, a dose of 0.3 mg kg^−1^ is severely toxic [7]. AMAs are potent inhibitors of RNA polymerase II, with bioactivity resistant to heat, cold, or acid inactivation. The typical distributions of α-AMA, β-AMA, and γ-AMA in a Death cap (*Amanita phalloides)* mushroom are approximately 43%, 43% and 14%, respectively [8,9]. A single dried mushroom typically contains around 1–2 mg g^−1^ of α-AMA [8,10,11].

The most common method for the detection of AMAs extracted from mushrooms is liquid chromatography (LC), coupled with UV detection or mass spectrometry (MS) [8,12,13,14]. Although these methods are sensitive and provide a high resolution of individual analytes, they are time-consuming and require expensive, laboratory-based instrumentation and highly trained personnel to interpret the results. In contrast, immunoassays are faster, can be field portable, and require less sophisticated instrumentation. The only commercially available antibody-based assay for AMA detection for research purposes is the Bühlmann assay [15]. This assay relies on a polyclonal antibody (pAb), which is a limited supply. Once the supply of antibody is depleted, the assay will have to be reevaluated for sensitivity and selectivity using a newly produced pAb. Since monoclonal antibodies (mAbs) are produced by a hybridoma cell line derived from a single cell, they overcome this supply limitation and have little or no batch-to-batch variability. Similarly, recombinant antibodies can be produced in large quantities, while preserving the monoclonality of the binding domain. Assays utilizing mAbs or recombinant antibodies are thus more desirable for long-term consistency and can be scaled-up for test kit manufacture. To our knowledge, only a few mAbs to AMAs have been described, and only one has been used for analytical detection [16,17,18].

Regardless of the method used to detect the toxin, extraction of the AMA is required before identification. Over the years, the extraction procedure has been streamlined from 24 h [8,10,19] to one hour [12,14,16,20]. Most of these methods have utilized an extraction solution consisting of methanol, acid, and water. Results from a latter study using a one hour extraction reported levels of α-AMA to be 0.88–1.33 mg g^−1^ dry weight [12], while earlier studies using the 24 hour extraction reported comparable levels of 0.75–2.8 mg g^−1^ dry weight [8,10] for the same species. Despite potential differences in the ages of mushrooms studied, these consistencies across studies suggest that extraction efficiency is not compromised with shortened extraction times. In addition, the historical methods use a combination of methanol, acid, and water to facilitate AMA extraction. Antibody-based immunoassays are often not compatible with large amounts of organic solvents or acidic solutions. Given the water solubility of AMAs, we hypothesized that a water-based AMA extraction would be sufficient for immunoassay detection.

The aim of this study was to utilize our previously reported immunogen, a periodate-oxidized form of α-AMA conjugated to the keyhole limpet hemocyanin (PERI-AMA-KLH) [20], to generate mouse mAbs. Then, we sought to use those mAbs to develop a sensitive and selective immunoassay for AMA detection from mushrooms. In this report, we describe and characterize novel anti-AMA mAbs and detail their performance in an indirect competitive inhibition enzyme-linked immunosorbent assay (cELISA). We compare the performance of this immunoassay for the detection of AMAs from mushrooms using difference extraction solutions. A sensitive detection assay for AMAs, combined with a rapid and simple toxin extraction method, would be a highly useful tool for the determination of AMA presence in wild mushrooms.

## 2. Results

### 2.1. Monoclonal Antibody Production

**Figure 2:**
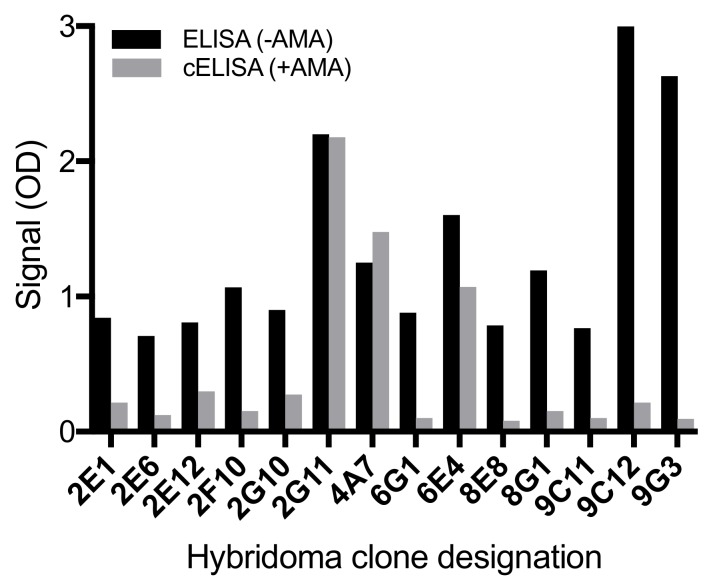
Hybridoma clone supernatants screened by indirect enzyme-linked immunosorbent assay (ELISA) (black bars) and by indirect competitive ELISA (gray bars). The cELISAs were completed using 100 ng mL^−1^ of α-amanitin as the competing analyte.

Mouse mAbs to AMAs were generated using the immunogen PERI-AMA-KLH [20]. Following the screening of the fusion plates, there were 14 positive cultures (optical density > 0.7), of which 12 cultures exhibited substantial signal reduction (optical density decreased by 0.5 or greater) in the presence of 100 ng mL^−1^ α-AMA in cELISA (Figure 2). Only two (9C12 and 9G3) of these grew stably, and were cloned multiple times until every well of the cell culture plate with cell growth elicited a positive indirect ELISA response to the coating antigen, a periodate-oxidized form of α-AMA conjugated to bovine serum albumin (PERI-AMA-BSA). The resulting mAbs were AMA9G3 (American Type Culture Collection Accession number PTA-125922) and AMA9C12 (American Type Culture Collection Accession number PTA-125923). Both mAbs were isotype IgG_1_-possessing kappa light chains.

### 2.2. Cross-Reactivity and Sensitivity

**Figure 3:**
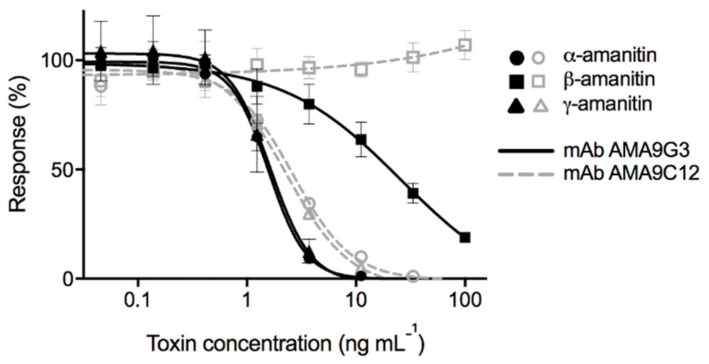
Standard cELISA inhibition curves for both monoclonal antibodies AMA9G3 (solid line) and AMA9C12 (dashed line) against toxins α-amanitin (circles), β-amanitin (squares), and γ-amanitin (triangles). R^2^ > 0.96 for the linear portion (including a minimum of three points) for every curve.

In order to determine how effective the assay would be in selectively detecting AMAs, a panel of cyclic peptides and smaller chemicals was tested. These included the bicyclic heptapeptides known as phallotoxins (phalloidin and phallacidin) also produced by *A. phalloides*, chemical toxins (psilocybin, muscimol, and ibotenic acid) produced by other mushrooms, and cyclic peptides (nodularin and microcystin-LR) produced by cyanobacteria. Of these analytes tested, AMA9G3 was competitively inhibited by all of the AMAs, α-AMA, β-AMA, and γ-AMA, while AMA9C12 was only competitively inhibited by α-AMA and γ-AMA (Table 1 and Figure 3). Both mAbs did not bind to any of the other compounds tested. 

The standard curves for the binding of both mAbs to the α-AMA, β-AMA, and γ-AMA toxins are shown in Figure 3. There is no reduction in signal response for AMA9C12 when tested against different concentrations of β-AMA, whereas, for AMA9G3, all three of the toxins did competitively inhibit at higher concentrations. The steepness of the curve indicated a small dynamic range for the assay because of the dramatic signal change produced by very small changes in toxin concentration, but simple sample dilutions could be performed to achieve a signal that fits within this range.

While both mAbs exhibited competitive inhibition from α-AMA and γ-AMA, AMA9G3 exhibited slightly higher sensitivity, with an IC_50_ of 1.57 ng mL^−1^ for α-AMA (Table 1 and Figure 3). For AMA9G3, the working range of detection (estimated as IC_30_ to IC_80_) of α-AMA is 0.7–3.1 ng mL^−1^, of γ-AMA is 0.5–2.4 ng mL^−1^, and of β-AMA is 3.6–129.1 ng mL^−1^. A conservative estimate for the limit of detection for α-AMA or γ-AMA with the AMA9G3 assay is 1 ng mL^−1^, accounting for the large (30%) variation in signal at low to no concentrations of toxin. Because of the propensity for samples (mushroom extracts) to contain all three AMAs, AMA9G3 was selected for use in the cELISAs for the extraction studies.

### 2.3. Kinetic Measurements

For all three antibodies (two mAbs from this study and one rabbit pAb #58 from our previous work), a final concentration of 10 nM was used in both the Equilibrium and Kinetics Injection studies. Table 2 shows the affinity (*K*_d_) and kinetic parameter (*k*_on_ and *k*_off_) values obtained for each antibody tested against α-AMA as the free ligand. *K*_d_ values of 10^−10^ M and lower indicate that these antibodies exhibit a high affinity for their target analyte (α-AMA). Given the similar affinity characteristics between the mAbs and the pAb, the major advantage of the mAbs over the pAb is their ability to produce a continuous supply of the same protein.

### 2.4. Mushroom Extraction

For the purposes of exploring the feasibility of performing simplified and rapid extractions, five different extraction solutions were tested. The commonly employed extraction using methanol and dilute acid was compared to extractions with more innocuous reagents, such as phosphates, tris, and Tween-20. The extraction solutions were tested on three different mushroom species. Both *A. phalloides* and *A. ocreata* are known to contain AMAs, while *A. gemmata* is known to not contain AMAs.

**Figure 4:**
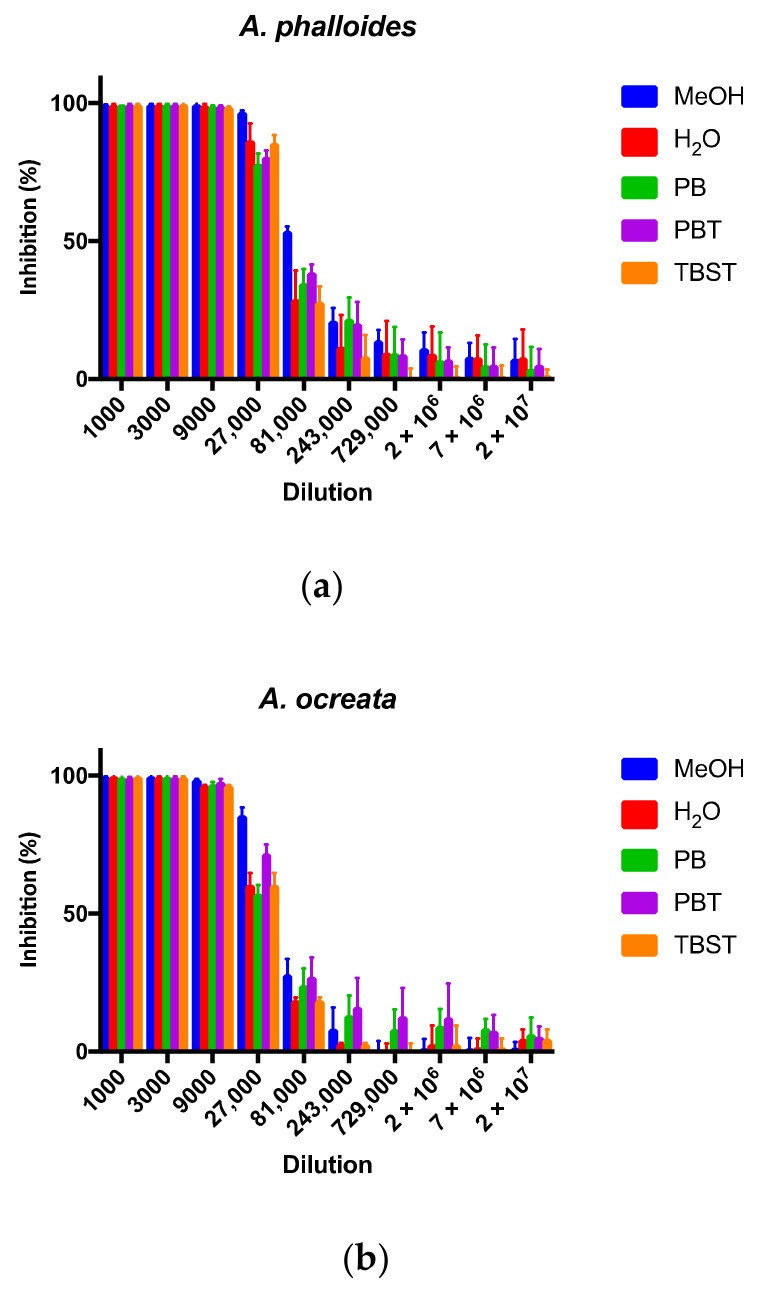

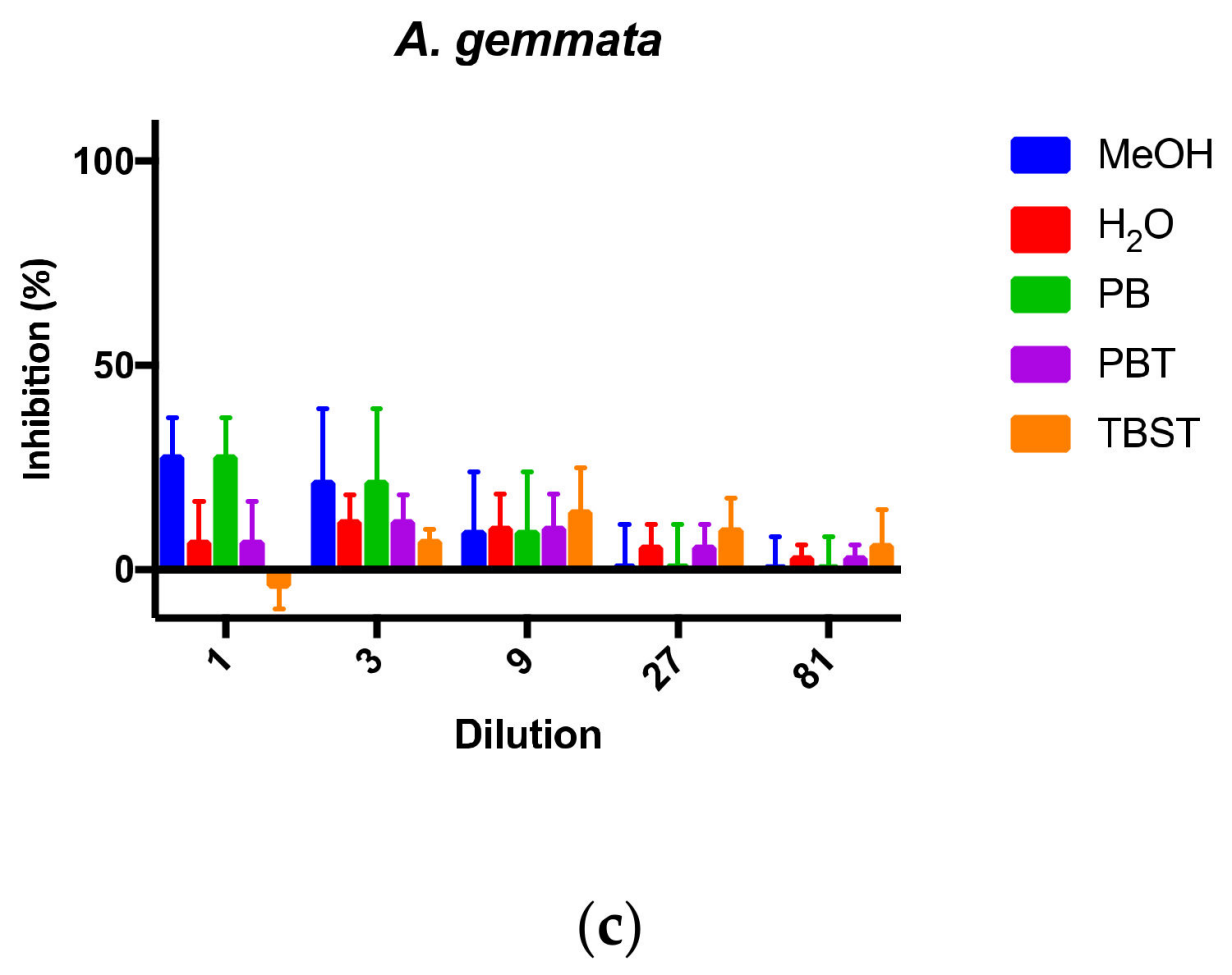
Representative cELISA inhibition profiles illustrating the amount of inhibition from the tested extraction conditions at varying dilutions of mushroom extracts obtained from (**a**) *Amanita phalloides*, (**b**) *A. ocreata*, and (**c**) *A. gemmata*. The extraction conditions were as follows: (1) MeOH: methanol: water: HCl for 1 h; (2) H_2_O: diH_2_O, 1 min, (3) PB: phosphate buffer, 1 min, (4) PBT: PB with Tween-20, 1 min, and (5) TBST: tris-buffered saline with Tween-20, 1 min.

AMAs were detected by this mAb-based cELISA in both of the extracts for the species known to contain AMAs (*A. phalloides* and *A. ocreata*) (Figure 4a,b). Detection is indicated by 100% inhibition at dilutions up to and including a 9000-fold dilution of the extract (Figure 4a,b). With increasing dilutions, it would be expected that the amount of inhibition would decrease as the AMA concentration decreases in a dose-dependent manner. Indeed, at dilutions greater than 9000-fold, decreased inhibition was observed for all extraction conditions.

There appeared to be very little difference between the different extraction conditions. The methanol extractions had a slightly higher inhibition at 27,000-fold and 81,000-fold dilutions for *A. phalloides* and the 27,000-fold dilution for *A. ocreata*. This may suggest that the methanol extractions extracted more AMAs than the aqueous extractions, since more inhibition equates to more toxin. However, the methanol extractions were carried out for 1 h, while the aqueous buffers were only carried out for 1 min. So, we cannot conclude whether time or composition contributed to the slight differences in extraction efficiency. However, because the differences were slight, we conclude that these aqueous buffers, with only a 1 min shaking step, were highly effective methods for AMA extraction.

With competitive-type assays, the signal intensity is the greatest when the free toxin to be detected is the lowest. In this case, low toxin levels are seen at the highest dilutions, and high signal intensities exhibit the most variation. With the data plotted as percent inhibition, higher variation was seen at higher dilutions (Figure 4). Thus, the background was estimated to be around 20–30% inhibition. Extracts from the non-toxin containing mushroom *A. gemmata* (Figure 4c) were intentionally tested at the more concentrated (e.g., 1- and 3-fold) dilutions, since no toxin was expected to be detected. The strong distinction between samples that exhibit 100% inhibition (i.e., with AMAs) and those without toxin demonstrated the ability of this mAb-based cELISA to selectively and sensitively detect AMAs from mushroom extracts.

## 3. Discussion

In this work, we report the generation of new anti-AMA mAbs (AMA9G3 and AMA9C12) using our previously synthesized immunogen (PERI-AMA-KLH) based on periodate oxidation of α-AMA. Unlike early reports of generating AMA-conjugated immunogens that exhibit toxicity [21,22], this immunogen did not cause any death in both mice (this study) or rabbits [20], corroborating the low toxicity observed by other investigators [23].

These mAbs exhibited high selectivity for AMAs, but the selectivity within AMAs varied from what was observed from our previously generated pAbs using the same immunogen [20]. The pAbs detected α-AMA, β-AMA and γ-AMA within a narrow range of IC_50_ values (2–3 ng mL^−1^). Conversely, both mAbs distinguish the AMAs differently, such that AMA9G3 detected α-AMA and γ-AMA (IC_50_ ≈ 1.6 ng mL^−1^), and, to a lesser extent, β-AMA (IC_50_ = 24 ng mL^−1^), while AMA9C12 detected only α-AMA and γ-AMA (IC_50_ = 2.3 and 2.7 ng mL^−1^, respectively). Similar to mAb AMA9C12, the commercially available, pAb-based cELISA detected only α-AMA and γ-AMA, and not β-AMA [15]. Another mAb, generated using a different coupling strategy to generate an α-AMA immunogen, produced mAbs with broad selectivity to α-AMA, β-AMA and γ-AMA (IC_50_ = 66, 97, and 163 ng mL^−1^, respectively) [16]. A single chain variable fragment of a mAb, produced by this same group, also exhibited broad selectivity to α-AMA, β-AMA and γ-AMA (IC_50_ = 77, 115, and 199 ng mL^−1^, respectively) [24].

Regarding total AMA extraction efficiency, the recovery obtained with all of these extraction methods is reputable. Previously reported α-AMA concentrations were approximately 1–2 mg of toxin per gram of dried mushroom [8,10,11], but this could vary depending on the location and age of the specimen [11]. Nonetheless, given our extraction ratio of 1 mL per 0.1 g of dried tissue, we would expect to recover 0.1–0.2 mg of α-AMA in 1 mL, as well as 0.1–0.2 mg of β-AMA and 0.05 mg of γ-AMA. This equates to extracts containing 10,000–45,000 ng mL^−1^ for total AMAs, which greatly exceeds our assay’s detection limit of 1 ng mL^−1^ for total AMAs. Furthermore, the detection of AMAs in the 27,000-fold dilution samples of these AMA-containing sample extracts provided evidence that we were obtaining a high recovery of AMA. Other investigators performed a second extraction following a 1 h extraction (using an acetonitrile solution) and did not recover any detectable amounts of residual toxin in the second extract [14]. While most of the AMAs appear to be easily extracted, further work is needed to determine the recovery coefficient for the rapid extraction procedure.

Furthermore, none of these tissue samples were macerated prior to extraction for any of the solutions tested. Maceration of dried mushroom samples increases exposure of the researcher to the toxin-containing dust. In our previous work, and that of many others, the mushroom tissue was ground to a powder [11,12,13,14,19,20,25]. In this study, however, we did not grind the samples and still achieved sufficient toxin extraction, suitable for cELISA detection. 

The sensitivity of our mAb-based cELISA permitted the detection of as little as 1 ng mL^−1^ of total AMAs in simple, water-based extracts obtained from dried mushrooms known to contain AMAs. Most LC methods achieve a detection limit of approximately 10 ng mL^−1^ for each individual AMA [8,14]. The amount of tissue required for an extraction can be as little as 50 mg for LC instrumental techniques, whereas the antibody-based detection methods could conceivably utilize sub-µg amounts of material using a simple, water-based extraction. The sensitivity, speed, and throughput of this mAb-based immunoassay for the detection of AMAs provides a simplified strategy for the evaluation of these toxins in wild mushrooms to characterize their occurrence and geographic distribution.

## 4. Materials and Methods

### 4.1. Immunization and Antibody Production

The Institutional Animal Care and Use Committee of the United States Department of Agriculture, Western Regional Research Center approved the experimental procedures used in these studies (protocol #16-1). Three 6-week-old female BALB/c mice were immunized by intraperitoneal injection (i.p.) of 100 μL of a 1:1 Sigma Adjuvant System (Sigma-Aldrich, St. Louis, MO, USA) containing 50 µg of PERI-AMA-KLH [20]. Two subsequent booster immunizations were administered i.p. at 2-week intervals using 20 µg of PERI-AMA-KLH in Sigma Adjuvant System. Serum were collected one week after the third immunization. Another two booster immunizations were performed four months later, two weeks apart, and serum was collected one week after this round of immunizations. After determining by indirect ELISA that the antibody response was still elevated to this target immunogen, a final booster immunization containing 10 µg of PERI-AMA-KLH in saline was administered i.p., four days prior to being euthanized and cell fusion.

### 4.2. ELISA Procedure

For serum antibody screening, black 96-well microtiter plates (Nunc, Thermo Fisher Scientific, Waltham, MA, USA) were coated at 1 μg mL^−1^ with PERI-AMA-BSA for 1 h at 37 °C in carbonate buffer (0.05 M carbonate-bicarbonate, pH 9.6). Then, the plates were blocked for 1 h at 37 °C with 3% non-fat dry milk in tris-buffered saline with 0.05% Tween-20 (TBST). After incubation for 1 h at 37 °C, TBST was removed and serum was loaded at a dilution of 1:100 in TBST and serially diluted. After another incubation for 1 h at 37 °C, plates were washed three times with TBST. Plates were then loaded with a secondary horse radish peroxidase labeled goat-anti-mouse antibody (Sigma) at 1:5000 in TBST and incubated for 1 h at 37 °C. Plates were washed and loaded with SuperSignal West Pico Chemiluminescent substrate (Fisher), incubated for 3 min, and then luminescent counts were recorded on a Victor^3^ Multilabel Counter (PerkinElmer, Waltham, MA, USA). 

### 4.3. Monoclonal Antibody Production and Screening

The cell fusion and expansion procedures were completed as previously described [26]. The screening of the cell culture plates following cell fusion, in particular the use of an indirect cELISA, was carried out as previously described with minor modifications [27]. The screening process was the same, but the reagents used were changed. Briefly, wells of clear-bottom microtiter plates coated with PERI-AMA-BSA were pre-loaded with 50 µL/well of either TBST for noncompetitive screening or α-AMA at 100 ng mL^−1^ for competitive screening. Plates were incubated for 1 h at 37 °C, washed, and then incubated with a secondary antibody, as described for the direct screening. After incubation and washing, antibody activity was visualized using Enhanced K-Blue Substrate (Neogen, Lexington, KY, USA) and read on a VersaMax Microplate Reader (Molecular Devices, San Jose, CA, USA). 

Hybridomas from wells exhibiting a significant reaction to the presence of α-AMA (i.e., a reduction in signal intensity) were selected for clonal expansion. Cells were cloned by limiting dilution, repeated until every well with cell growth presented positive activity via ELISA. MAbs were purified from the cell culture supernatant on a Protein G Sepharose affinity column (GE Healthcare Life Sciences, Pittsburgh, PA, USA), eluted with 0.1 M glycine-HCl, pH 2.7. Purified protein was extensively dialyzed against the phosphate buffered saline (PBS; 10 mM phosphate, 138 mM NaCl, 2.7 mM KCl, pH 7.4) and then stored at −20 °C until further use. Antibody protein concentrations were determined on a NanoDrop Lite Spectrophotometer (Thermo). Antibody isotyping was completed using an IsoStrip Mouse Monoclonal Antibody Isotyping Kit (Roche, Indianapolis, IN USA), following the manufacturer’s protocol. Purified mAbs were titrated by indirect ELISA to determine the concentration of the antibody at half of the maximal signal. This determined concentration was used as the working concentration of the antibody for the cELISAs, to evaluate antibody cross-reactivity.

### 4.4. Antibody Characterization: Cross-Reactivity

Indirect cELISAs were completed using a panel of inhibitors to determine the selectivity of the mAbs. The cELISA procedure was nearly the same as that described for the serum screening, except for the addition of inhibitors (50 µL), which were mixed with 50 µL of the antibody solution during the primary antibody incubation step. The inhibitors tested were α-AMA (≥90%, Enzo Life Sciences, Farmingdale, NY, USA), β-AMA (≥90%, Enzo), γ-AMA (≥90%, Enzo), microcystin-LR (≥95%, Enzo), nodularin (≥95%, Enzo), phalloidin (>90%, Enzo), phallacidin (≥85%, Sigma), pysilocybin (>99%, Cerilliant, Round Rock, TX, USA), muscimol (>99%, Abcam, Cambridge, MA, USA), ibotenic acid (>98%, Abcam). Each analyte stock was dissolved in dH_2_O, then serially diluted into TBST, starting at the highest concentration of ,000 ng mL^−1^, and assessed in triplicate. Data were analyzed using a 4-parameter logistic equation (GraphPad Prism 7 Software, La Jolla, CA, USA) to determine the concentration of inhibition at half of the maximal signal (IC_50_). Cross-reactivity (%) was calculated as follows: (IC_50_ α-AMA)/(IC_50_ test inhibitor) × 100.

### 4.5. Antibody Characterization: Kinetic Measurements

All KinExA experiments were performed on a KinExA 3200 with Autosampler (Sapidyne Instruments, Boise, ID, USA) and data were analyzed using KinExA Pro software provided by Sapidyne. Affinity values (*K*_d_) utilized were their template protocol for an Equilibrium Experiment and kinetic parameters were determined using the Kinetics Injection method. Flow rates and volumes used were the default settings defined in the software. 

Polymethylmethacrylate particles (aliquots of 200 mg, Syringa Labs, Boise, ID, USA) were adsorption coated with 30 µg of BSA-AMA-PERI in 1 mL of carbonate buffer for 1 h at room temperature with end-over-end rotation. The particles were blocked with a solution of 1% BSA (Sigma) in PBS for 1 h at room temperature with end-over-end rotation, and stored at 4 °C for no more than one week before use. The diluent for all reagents was PBS containing 1% BSA. Three antibodies were evaluated—two mouse mAbs (AMA9G3 and AMA9C12) generated from this study and one rabbit pAB #58 generated from the previous study [20]. The secondary antibody used for the mouse mAb experiments was DyLight650 labeled anti-mouse Ig (Fisher) (used at 0.5 µg mL^−1^) and the secondary antibody used for the rabbit pAb experiments was AlexaFluor647 labeled anti-rabbit Ig (Jackson Immunoresearch, West Grove, PA, USA) (used at 0.25 µg mL^−1^).

Signal test runs were completed on each antibody to determine the amount of antibody needed to generate the appropriate signal change (1 Δv). Then, for the Equilibrium experiments, antibody was prepared at 2× this concentration and then mixed with an equal volume of a solution containing α-AMA diluted 2-fold, ranging from 300 ng mL^−1^ (326 nM) to 9.2 pg mL^−1^ (10 pM) of final concentrations, including one sample with no α-AMA and one sample containing only diluent. For the Kinetics Injection experiments, the same 2× antibody concentration was used, along with solutions containing α-AMA diluted 2-fold, ranging from 920 ng mL^−1^ (1000 nM) to 1.8 ng mL^−1^ (2 nM). The Equilibrium and Kinetics Injection experiments were completed in duplicate. 

### 4.6. Mushroom Extraction

Whole mushroom specimens were identified, dried, and provided by expert mycologists. The specimens included two that were known to contain AMAs, *A. phalloides* and *A. ocreata*, and one that was known to not contain AMAs, but was from the same genus, *A. gemmata*. Small portions of the specimens were weighed (~100–200 mg) and then placed into a 15 mL Falcon tube containing one of the five extraction buffers: (1) methanol (methanol:water:0.01 N HCl, 5:4:4, v:v:v), (2) diH_2_O, (3) phosphate buffer (PB; 0.1 M, pH 7.6), (4) PB with Tween-20 (PBT), or (5) TBST at a ratio of 1 mL per 100 mg tissue. The samples that were extracted with the methanol buffer were shaken for 1 h at room temp and then centrifuged at 1000× *g* for 10 min. Aliquots of the supernatant were drawn off, diluted in TBST as necessary, and assessed by indirect cELISA. The samples in diH_2_O, PB, PBT, or TBST were briefly shaken by hand for 1 min. Immediately after shaking, a 50 µL aliquot of the liquid phase was drawn off, diluted in TBST as necessary, and assessed by indirect cELISA, as described earlier. At least two individual mushrooms from each species were extracted, and extractions for each extraction condition were completed in duplicate.

## Figures and Tables

**Table 1:** Cross-reactivity (%) of compounds found in associated mushrooms or structurally related compounds.

	mAb AMA9G3		mAb AMA9C12		Bühlmann Assay [15]
Toxin	IC_50_ (ng mL^−1^)	Cross Reactivity (%)	IC_50_ (ng mL^−1^)	Cross Reactivity (%)	Cross Reactivity (%)
α-amanitin	1.57 ± 0.07	100	2.66 ± 0.18	100	100
β-amanitin	24.2 ± 6.2	6.5	>1000	<0.3	0.1
γ-amanitin	1.63 ± 0.21	96	2.3 ± 0.31	115	90
phalloidin	>1000	<0.3	>1000	<0.3	Not detected
phallacidin	>1000	<0.3	>1000	<0.3	Not detected
psilocybin	>1000	<0.3	>1000	<0.3	Not determined
microcystin-LR	>1000	<0.3	>1000	<0.3	Not determined
nodularin	>1000	<0.3	>1000	<0.3	Not determined
ibotenic acid	>1000	<0.3	>1000	<0.3	Not determined
muscimol	>1000	<0.3	>1000	<0.3	Not determined

**Table 2:** Affinities (*K*_d_) and kinetic parameters (*k*_on_ and *k*_off_) for antibodies binding to α-amanitin measured by KinExA.

Antibody	*K*_d_ (M)	*k*_on_ (M^−1^ s^−1^)	*k*_off_ (s^−1^)
rabbit pAb 58	3.5 × 10^−11^	4.1 × 10^6^	1.4 × 10^−4^
mAb AMA9G3	6.4 × 10^−11^	4.7 × 10^7^	3.0 × 10^−3^
mAb AMA9C12	9.3 × 10^−10^	1.7 × 10^7^	1.4 × 10^−2^
